# USP48 Governs Cell Cycle Progression by Regulating the Protein Level of Aurora B

**DOI:** 10.3390/ijms22168508

**Published:** 2021-08-07

**Authors:** Ainsley Mike Antao, Kamini Kaushal, Soumyadip Das, Vijai Singh, Bharathi Suresh, Kye-Seong Kim, Suresh Ramakrishna

**Affiliations:** 1 Graduate School of Biomedical Science and Engineering, Hanyang University, Seoul 04763, Korea; ainsleyantao@gmail.com (A.M.A.); kaminikaushal10@gmail.com (K.K.); smdpdas6@gmail.com (S.D.); bharathi.suri@gmail.com (B.S.); 2 Department of Biosciences, School of Science, Indrashil University, Rajpur, Ahmedabad 382740, Gujarat, India; vijai.singh@indrashiluniversity.edu.in; 3 College of Medicine, Hanyang University, Seoul 04763, Korea

**Keywords:** aurora kinase, CRISPR/Cas9, DUBs, gene knockout, mitotic regulator, post-translational modifications

## Abstract

Deubiquitinating enzymes play key roles in the precise modulation of Aurora B—an essential cell cycle regulator. The expression of Aurora B increases before the onset of mitosis and decreases during mitotic exit; an imbalance in these levels has a severe impact on the fate of the cell cycle. Dysregulation of Aurora B can lead to aberrant chromosomal segregation and accumulation of errors during mitosis, eventually resulting in cytokinesis failure. Thus, it is essential to identify the precise regulatory mechanisms that modulate Aurora B levels during the cell division cycle. Using a deubiquitinase knockout strategy, we identified USP48 as an important candidate that can regulate Aurora B protein levels during the normal cell cycle. Here, we report that USP48 interacts with and stabilizes the Aurora B protein. Furthermore, we showed that the deubiquitinating activity of USP48 helps to maintain the steady-state levels of Aurora B protein by regulating its half-life. Finally, USP48 knockout resulted in delayed progression of cell cycle due to accumulation of mitotic defects and ultimately cytokinesis failure, suggesting the role of USP48 in cell cycle regulation.

## 1. Introduction

The proper progression of the cell cycle is orchestrated by a highly coordinated series of molecular events to ensure faithful DNA replication and equal chromosomal segregation into two daughter cells [1]. Aurora B (AURKB) belongs to the family of serine/threonine protein kinases that play key roles in regulating the mammalian cell division cycle [2]. This family also includes Aurora A which functions in the centrosome [3] and Aurora C that regulates meiosis and mitotic events during early embryogenesis [4,5]. Aurora B forms the kinase module of the highly conserved chromosomal passenger complex (CPC), which is a “master controller” of the cell cycle [6]. The CPC complex additionally contains a localization module comprising of the scaffolding protein inner centromere protein (INCENP), Survivin, and Borealin [6].

Aurora B plays a role in regulating almost every stage of mitosis including the condensation, bi-orientation, and segregation of chromosomes, formation of the spindle checkpoints, and cytokinesis [2,7]. Aurora B along with the other CPC proteins ensures the proper segregation of chromosomes by destabilizing incorrect, erroneous kinetochore-microtubule attachments [8,9]. Unattached kinetochores also trigger the activation of spindle assembly checkpoint control (SAC) that delays the progression of the cell cycle [9]. Aurora B inhibits premature SAC silencing by preventing the removal of SAC proteins from unattached kinetochores. Interestingly, not just the loss, but also an increase in Aurora B activity leads to chromosomal segregation errors and poses a threat for cell viability [10]. Given the importance of Aurora B in regulating the cell cycle, the dysregulation of Aurora B protein levels was hypothesized to provide a proliferative advantage to cancer cells [11,12]. Elevated Aurora B kinase levels were reported in various malignancies including thyroid carcinoma [13], non-small cell lung cancer [14], breast cancer [15], testicular germ cell tumors [16], human seminoma [17], mesothelioma [18], glioblastoma [19], prostate [20], and colon cancer [21].

Thus, understanding regulatory mechanisms that control Aurora B protein levels during key cell cycle events has gained significant attention. Post-translational modifications (PTM) are critical in controlling the functions of Aurora B along with other CPC complex proteins. Aurora B was reported to be regulated by SUMOylation [22], acetylation/deacetylation [23], phosphorylation [24], and poly(ADP-ribosyl)ation [25]. Among several PTMs of Aurora B, ubiquitination/deubiquitination is reported as a major regulator of Aurora B protein turnover [26,27]. Numerous groups have previously reported the importance of Aurora B ubiquitination in regulating its dynamic functions during mitosis by affecting its localization and degradation [28,29,30,31,32]. Deubiquitinating enzymes (DUBs) reverse the ubiquitination process by cleaving Ub moieties from target proteins, thereby regulating key cellular functions [33]. So far, DUBs such as USP13 [34], USP14 [35] and USP35 [36] that reverse the levels of Aurora B ubiquitination were reported.

In this study, we used a CRISPR/Cas9-based DUB targeting sgRNA kit to specifically knockout DUBs belonging to the USP sub-family of proteins [37]. The loss-of-function of individual DUBs that resulted in the reduction of Aurora B protein expression helped us to identify USP48 as a potential DUB regulating Aurora B. We demonstrated that USP48 binds to Aurora B protein and extends its half-life by its deubiquitinating activity. Furthermore, the depletion of USP48 resulted in cells showing delayed progression through the cell cycle due to the accumulation of mitotic defects and, ultimately, cytokinesis failure. Taken together, our data suggest that USP48 plays a critical role in maintaining the steady-state levels of Aurora B by its deubiquitinating activity, thereby ensuring faithful mitotic progression.

## 2. Results

### 2.1. CRISPR-Based Genome-Scale Screening of USP Sub-Family Proteins Exhibiting Reduction of Aurora B Protein Levels

**Figure 1:**
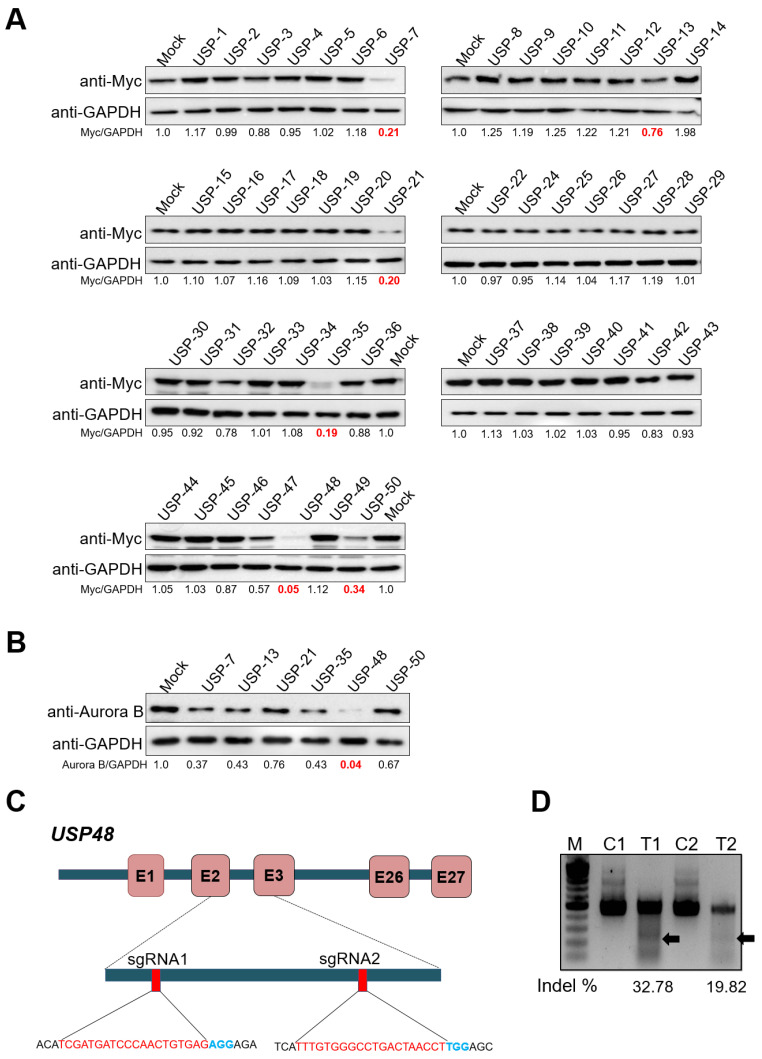
CRISPR-based genome-scale screening of USP sub-family proteins exhibiting a reduction in Aurora B protein levels. (**A**) Screening for DUBs regulating Myc-Aurora B was performed using a CRISPR-Cas9-based DUBKO sgRNA kit. HEK293 cells were transfected with Myc-Aurora B and the indicated sgRNAs along with Cas9. Equal concentrations of proteins from DUBKO HEK293 cell lysates were subjected to Western blot analyses. The protein band intensities were estimated using ImageJ software with reference to the GAPDH control band for each individual sgRNA (Myc-Aurora B/GAPDH). The loss of USPs leading to the downregulation of the Aurora B protein level is marked in red. (**B**) The putative DUBs USP7, USP13, USP21, USP35, USP48, and USP50, which potentially regulate Aurora B protein levels, were transfected in HeLa cells along with Cas9. The protein band intensities were estimated using ImageJ software with reference to the GAPDH control band for each individual sgRNA (Aurora B/GAPDH). The protein band intensity of HeLa cells transfected with sgRNA targeting *USP48* showed the highest reduction in the endogenous Aurora B is represented in red. (**C**) Schematic of RNA-guided engineered nuclease targeting the sequences in exon 2 and exon 3 of the human *USP48* gene using sgRNA1 and sgRNA2, respectively. PAM sequences are represented in blue, while the sgRNA target sequences are represented in red. (**D**) T7E1 assays were performed in HEK293 cells to determine the cleavage efficiency of sgRNA1 (T1) and sgRNA2 (T2). The cleaved band intensity (indicated by arrow) obtained from the T7E1 assay was estimated using ImageJ software and represented as indel percentage (indel %). Scrambled sgRNA-transfected cells were used as control cells (C). A marker is shown for size reference.

We first aimed to identify potential DUBs responsible for regulating levels of Aurora B protein by Western blot analysis. To this end, we used our recently developed DUB knockout sgRNA kit consisting of sgRNAs targeting USP sub-family proteins [37]. We investigated significant changes in the protein levels of ectopically expressed Myc-Aurora B by individually transfecting sgRNAs targeting DUBs in HEK293 cells. Our screening system identified several DUBs, including USP7, USP13, USP21, USP35, USP48, and USP50, showing reductions in the expression of Myc-Aurora B as compared to the mock-transfected cell lysates (Figure 1A). We further validated the putative positive DUB candidates by checking their effects on endogenous Aurora B protein levels in HeLa cells. Among the putative DUBs (USP7, USP13, USP21, USP35, USP48, and USP50), the knockdown of USP48 showed a significant reduction in endogenous Aurora B than other tested DUBs (Figure 1B). The regulatory effect of USP48 on Aurora B protein was similar to that of USP35, recently identified as a deubiquitinase for Aurora B protein [36].

### 2.2. USP48 Regulates Aurora B Protein Stability

**Figure 2:**
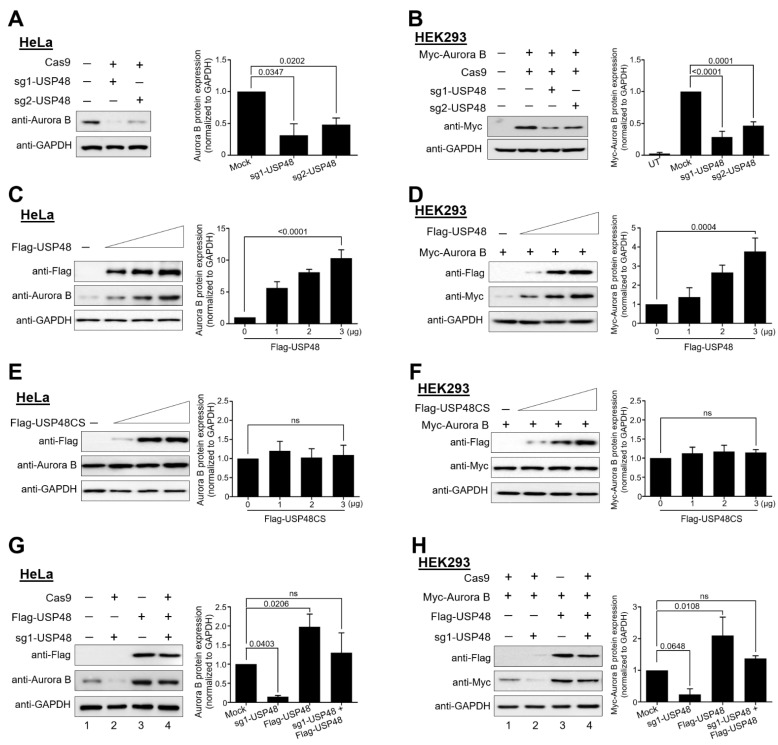
USP48 regulated Aurora B protein stability. The efficiencies of sgRNA1 and sgRNA2, targeting the *USP48* gene, in regulating the (**A**) endogenous or (**B**) exogenous Aurora B protein stabilization was determined by transfecting in HeLa and HEK293 cells, respectively, along with Cas9. (**C**) HeLa cells were transfected with an increasing amount of Flag-USP48 (0,1,2, and 3 µg), and the endogenous expression of Aurora B protein was analyzed by Western blot. (**D**) HEK293 cells were transfected with an increasing amount of Flag-USP48 (0,1,2, and 3 µg), along with a constant amount of Myc-Aurora B (0.5 µg), and the expression of Myc-Aurora B protein was analyzed by Western blot. (**E**) HeLa cells were transfected with increasing amounts of catalytically inactive Flag-USP48CS (0,1,2, and 3 µg), and the endogenous expression of Aurora B protein was analyzed by Western blot. (**F**) HEK293 cells were transfected with increasing amounts of Flag-USP48CS (0,1,2, and 3 µg), along with a constant amount of Myc-Aurora B (0.5 µg). The expression of Myc-Aurora B protein was analyzed by Western blot. Reconstitution experiments were performed to validate the specificity of USP48 for the stabilization of (**G**) endogenous or (**H**) ectopically expressed Aurora B in HeLa and HEK293 cells, respectively. All the experiments were performed in triplicates and band intensities were estimated using ImageJ software with reference to the GAPDH control band and graphically represented. One-way ANOVA followed by Tukey’s post hoc test was used and the *p* values are represented on the figures (ns = non-significant).

To further investigate the role of USP48 in regulating Aurora B protein stability, we designed two sets of sgRNAs targeting exon 2 and exon 3 of *USP48,* as depicted in Figure 1C. The gene disruption efficiency of sgRNA1 showed a higher indel percentage than sgRNA2 by T7E1 assay (Figure 1D). The effect of sgRNA1 and sgRNA2 targeting *USP48* showed reductions in endogenous Aurora B protein levels in HeLa cells (Figure 2A). Moreover, sgRNA1 targeting *USP48* showed a significant reduction in the protein level of ectopically expressed Myc-Aurora B compared to that of sgRNA2 (Figure 2B).

We next analyzed the steady-state levels of endogenous Aurora B and Myc-Aurora B proteins upon the dose-dependent overexpression of Flag-USP48 or its catalytically inactive form Flag-USP48 C98S (Flag-USP48CS). We observed a steady increase in the protein expression of endogenous Aurora B in HeLa cells (Figure 2C) as well as ectopically expressed Myc-Aurora B in HEK293 cells (Figure 2D) upon the dose-dependent increase in Flag-USP48. This stabilization effect was not observed upon the dose-dependent increase in Flag-USP48CS on both endogenous Aurora B in HeLa cells (Figure 2E) as well as on ectopically expressed Myc-Aurora B in HEK293 cells (Figure 2F), indicating that USP48 might act as a protein stabilizer of both endogenous and exogenous Aurora B through its deubiquitinating activity.

Next, we validated the specificity of USP48 stabilization of endogenous Aurora B and ectopically expressed Aurora B protein by performing reconstitution experiments in HeLa and HEK293 cells. Our results confirmed the reduction in endogenous Aurora B protein levels as the effect of sgRNA1 targeting *USP48* (Figure 2G, lane 2) was recovered by the ectopic expression of Flag-USP48 (Figure 2G, lane 4). Similarly, the reduction in Myc-Aurora B protein upon the knockdown by sgRNA1 targeting *USP48* (Figure 2H, lane 2) was reversed following reconstitution with Flag-USP48 (Figure 2H, lane 4).

### 2.3. USP48 Deubiquitinates Aurora B Protein and Extends Its Half-Life

**Figure 3:**
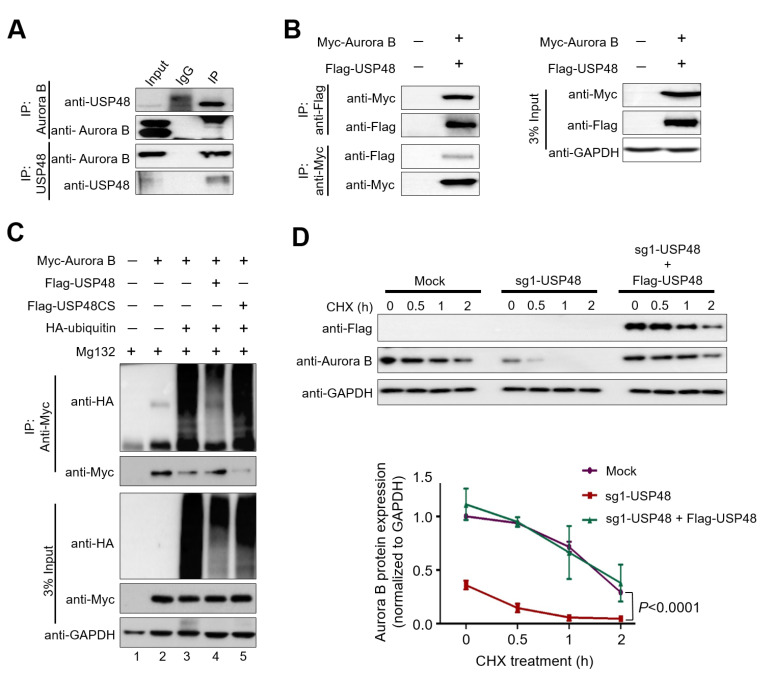
USP48 interacts, deubiquitinates, and extends Aurora B protein half-life. (**A**) The endogenous interactions between the USP48 and Aurora B proteins were examined by immunoprecipitation (IP) experiments in HeLa cells. Cell lysates from HeLa cells were immunoprecipitated and immunoblotted with specific USP48 or Aurora B antibodies as indicated. (**B**) Exogenous IP experiments to demonstrate the interaction between Myc-Aurora B and Flag-USP48 were performed in HEK293 cells. Samples were immunoprecipitated and immunoblotted using either anti-Flag or anti-Myc antibodies as indicated. GAPDH was used as a loading control. (**C**) Myc-Aurora B and HA-ubiquitin were co-transfected along with either Flag-USP48 or Flag-USP48CS in HEK293 cells. The ubiquitination level of Aurora B protein was confirmed by performing IP with an anti-Myc antibody and immunoblotting with the anti-HA (ubiquitin) and anti-Myc antibody as indicated. (**D**) The cycloheximide assay was performed to demonstrate the half-life of endogenous Aurora B protein in cells transfected with mock control, sgRNA1 targeting *USP48*, and upon the reconstitution with Flag-USP48 in sgRNA1 targeted *USP48* HeLa cells. Experiments were performed in triplicates and band intensities were estimated using ImageJ software with reference to the GAPDH control band and graphically represented. Two-way ANOVA followed by Tukey’s post hoc test was used and the *p* values are represented.

Following the results demonstrating the ability of USP48 to stabilize the endogenous and exogenous Aurora B proteins, we investigated whether both these proteins could interact. To this end, we performed endogenous immunoprecipitation (IP) experiments in HeLa cells. IP using endogenous USP48 antibodies was found to co-immunoprecipitate endogenous Aurora B and vice versa in HeLa cells (Figure 3A), indicating that USP48 interacts with endogenous Aurora B. Additionally, we performed exogenous IP experiments by co-transfecting Flag-USP48 and Myc-Aurora B in HEK293 cells. We found that Flag-USP48 could co-immunoprecipitate with Myc-Aurora B and vice versa (Figure 3B).

Aurora B protein was previously reported to be regulated by the ubiquitin-proteasome system [27,34,36]. To analyze the effect of USP48 on the ubiquitination status of Aurora B protein, we transiently overexpressed Flag-USP48 or its catalytic mutant Flag-USP48CS along with Myc-Aurora B and HA-Ubiquitin in HEK293 cells. Flag-USP48 overexpression reduced the polyubiquitination smear attached to Myc-Aurora B protein as compared to the mock-transfected cells (Figure 3C, lane 4 vs. lane 3), whereas the catalytic mutant Flag-USP48CS did not alter the ubiquitination status of Aurora B protein (Figure 3C, lane 5 vs. lane 3).

Furthermore, we performed the cycloheximide (CHX) chase assay to block the synthesis of new peptides in HeLa cells to determine whether the modulation of USP48 levels directly impacted Aurora B protein half-life. We analyzed the endogenous Aurora B protein expression in HeLa cells transfected with either mock, sgRNA1 targeting *USP48*, and upon the reconstitution with Flag-USP48 in sgRNA1 targeting *USP48* transfected HeLa cells which were treated with CHX for the indicated time-course (0 h, 0.5 h,1 h, and 2 h) (Figure 3D). We found that the depletion of USP48 lead to a reduction in the half-life of Aurora B, while overexpression of USP48 using Flag-USP48 rescued the half-life of Aurora B (Figure 3D), indicating that USP48 acts as an Aurora B stabilizer.

### 2.4. USP48 Influences Cell Cycle Progression

**Figure 4:**
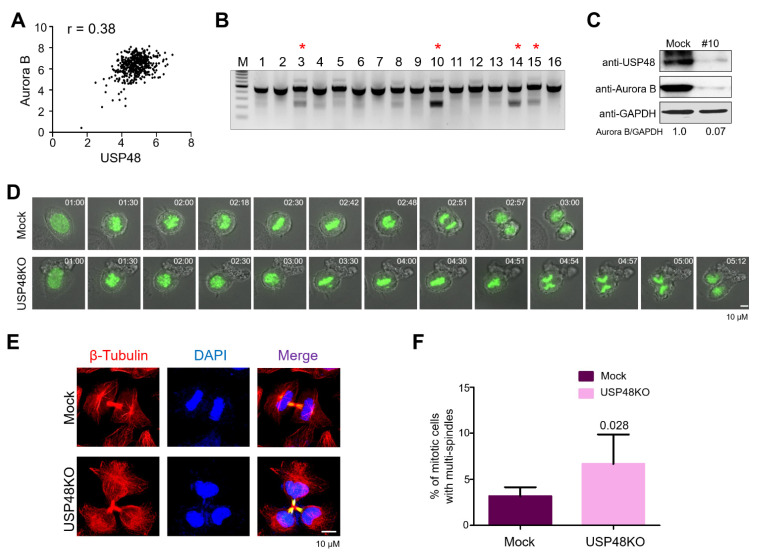
USP48 influences cell cycle progression. (**A**) A scatterplot demonstrating the correlation between *USP48* and *Aurora B* mRNA levels was plotted. Pearson correlation analysis (r) was used to quantify the relationship between *USP48* and *Aurora B* (*n* = 455). (**B**) Single-cell-derived USP48KO colonies were screened using the T7E1 assay. Red asterisk represents the T7E1 positive USP48KO clones, i.e., USP48KO clones #3, #10, #14, and #15. (**C**) The effect of USP48 knockout on Aurora B protein expression was checked by Western blot analysis in USP48KO clone #10 upon M phase synchronization with 100 ng/mL Nocodazole for 18 h. GAPDH was used as a loading control. (**D**) Time-lapse microscopy of mock and USP48KO HeLa cells transfected with GFP-H2B and treated with thymidine and released into fresh medium to track cells undergoing mitosis. Time points are indicated in hh:mm format. Scale bar = 10 μM. (**E**) Microtubules were stained with β-Tubulin-Cy3 antibodies in mock and USP48KO HeLa cells. DAPI was used to stain the nuclei. Scale bar = 10 μM. (**F**) Graph representing the percentage of multinucleated cells in mock (3.166 ± 0.98), and USP48KO HeLa cells (6.667 ± 3.20) (*n* = 3, *p* = 0.028).

After demonstrating that the modulation of USP48 levels could regulate the Aurora B protein half-life, we attempted to analyze the correlation between these two proteins at the mRNA level using data from the Cancer Cell Line Encyclopedia (CCLE) database. We observed that the high scores for *USP48* mRNA expressions were directly proportional to the scores for *Aurora B* mRNA expression, suggesting a significant positive correlation between *USP48* and *Aurora B* mRNA for all the cancer cell lines tested (*n* = 455, *p* < 0.0001; r = 0.3807) (Figure 4A). Additionally, using The Cancer Genome Atlas (TCGA) database, we analyzed the mRNA expression levels of *USP48* and *Aurora B* in breast invasive carcinoma (BRCA), lung adenocarcinoma (LUAD), cervical squamous cell carcinoma (CESC) and prostate adenocarcinoma (PRAD) (Supplementary Figure S1). We observed a significant upregulation in the mRNA expression of both *USP48* and *Aurora B* in BRCA (Supplementary Figure S1A) and LUAD (Supplementary Figure S1B) when compared to their respective normal tissues indicating an association between *USP48* and *Aurora B* mRNA expression levels.

Given that *USP48* and *Aurora B* mRNA expression levels were positively correlated in cancer cells, we investigated whether the knockout of *USP48* could affect the functions of Aurora B during the cell cycle. To this end, we generated single-cell-derived USP48 knockout (USP48KO) clones in HeLa cells using the CRISPR/Cas9 system. HeLa cells transfected with plasmids encoding sgRNA1 targeting *USP48* and Cas9 were subjected to single-cell dilution by seeding into 96-well plates. Individual single-cell-derived clones were subjected to the T7E1 assay to analyze *USP48* gene disruption. T7E1-positive USP48KO clones #3, #10, #14 and #15 demonstrated disruption of *USP48* gene (Figure 4B). HeLa cells transfected with scrambled sgRNA along with Cas9 were subjected to single-cell dilution and considered as mock controls in our experiments.

It is already well documented that the expression of Aurora B varies during the progression of the cell cycle [2], prominent during the G2-M phase of the cell cycle [38,39]. As such, we synchronized cells using Nocodazole, a microtubule inhibitor, to inhibit mitosis and analyzed the protein expression of Aurora B in the presence or absence of USP48. We observed a decrease in endogenous Aurora B expression in USP48KO clone #10 HeLa cells as compared to mock cells that were synchronized at the mitotic phase (Figure 4C). Hereafter, USP48KO clone #10 (referred to as USP48KO) was used for all cell cycle studies.

**Figure 5:**
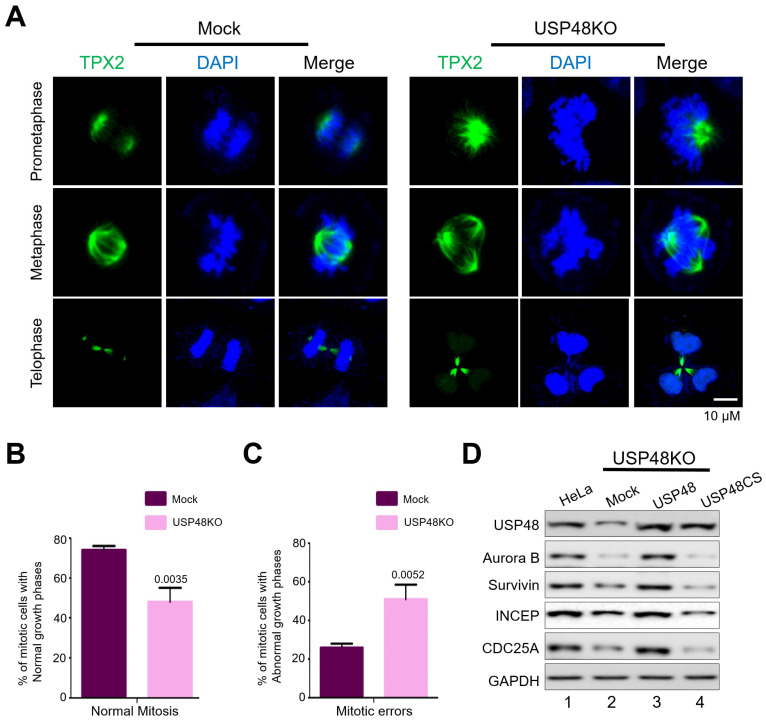
USP48 regulates Aurora B function during mitosis. (**A**) The spindle assembly factors in mock (left panel) and USP48KO (right panel) HeLa cells were stained with TPX2 antibodies. DAPI was used to stain the nuclei. Scale bar = 10 μM. Mitotic defects were observed upon depletion of USP48. The graph represents the percentage of cells exhibiting (**B**) normal mitotic phases in mock (74 ± 2), and USP48KO (48 ± 7) and (**C**) abnormal mitotic phases in mock (26 ± 2) and USP48KO (51 ± 7.55) (*n* = 3, *p* values are represented on each graph). (**D**) USP48KO HeLa cells (lane 2) were transfected with either Flag-USP48 (lane 3) or Flag-USP48CS (lane 4) and compared with wild-type HeLa cells (lane 1). Transfected cells were synchronized with 100 ng/mL Nocodazole for 18 h prior and analyzed by Western blot against the indicated antibodies.

To investigate the effect of loss of USP48 on mitotic progression, we performed live-cell imaging of USP48KO and mock HeLa cells expressing GFP-H2B. Loss of USP48 delayed mitotic timing as compared to the mock HeLa cells (Figure 4D, Supplementary movie 1). Further, microscopic analysis of immunostained microtubules in USP48KO HeLa cells exhibited a higher frequency of cytokinesis failures as compared with the mock HeLa cells (Figure 4E). USP48KO cells displayed three or more daughter cells forming multi-nuclear phenotypes (Figure 4E) in late telophase that are represented graphically as a percentage of mitotic cells with multi-spindles in mock, and USP48KO (*p* = 0.028, Figure 4F). Further evidence for cytokinesis failure was supported by staining the spindle assembly factors with TPX2 antibody to follow the mitotic progression. Depletion of USP48 resulted in increased mitotic defects, such as lagging chromosomes, misaligned chromosomes, and multipolar spindles, as compared to the mock (Figure 5A). Percentage of mitotic cells demonstrating normal growth phases in mock, and USP48KO, (*p* = 0.0035, Figure 5B); and the percentage of mitotic cells demonstrating abnormal growth phases in mock and USP48KO, (*p* = 0.0052, Figure 5C) HeLa cells are graphically represented.

We additionally validated the significance of Aurora B protein stabilization during the cell cycle by reconstituting USP48 in USP48-depleted HeLa cells. To this end, we transfected either Flag-USP48 or Flag-USP48CS in USP48KO cells and analyzed key genes associated with cell cycle progression. Interestingly, the reduction in protein expression of Aurora B upon depletion of USP48 correlated with a reduction in cell cycle-associated genes, including INCENP, Survivin and CDC25A, as compared to that of HeLa cells (Figure 5D, lane 2 vs. lane 1). The reduction in protein expression of Aurora B along with INCENP, Survivin and CDC25A was reversed upon the reconstitution of Flag-USP48 but not upon Flag-USP48CS reconstitution (Figure 5D, lane 3 and lane 4 vs. lane 1) in USP48KO HeLa cells, suggesting the importance of USP48 in regulating the cell cycle. Overall, our results suggest the importance of USP48 in regulating cell cycle progression via Aurora B protein stabilization.

## 3. Discussion

The ubiquitin–proteasome pathway plays a vital role in regulating the cell cycle [1]. Aurora B along with other components of the CPC complex, function actively during mitosis and were reported to be regulated by several PTMs during the cell cycle. Several DUBs were reported to modulate the cell cycle by regulating Aurora B proteins. For instance, the deubiquitinating activity of USP9X regulates proper targeting and association of Survivin and Aurora B proteins to the centromeres [40], USP39 regulates the mRNA splicing of Aurora B [41], while USP14 was reported to regulate the stability of Aurora B in leukemia cells [35]. USP35 was reported to regulate the stability of Aurora B by opposing its proteasomal degradation triggered by APC/C-Cdh1 ubiquitination [36]. Recently, the USP13 DUB has was also reported to associate with Aurora B and regulate its stability, especially before mitosis; this association is promoted by the phosphorylation of USP13 by Aurora B kinase [34].

Considering the importance of DUBs in regulating key processes during the cell cycle, we initiated this study by screening a panel of roughly 50 DUBs that may potentially regulate the Aurora B protein. We used our previously described DUB sgRNA library kit [37] based on the CRISPR/Cas9 system to knock down individual DUBs belonging to the USP family. The data we present here establishes USP48 as another DUB that can control the cell cycle by deubiquitinating the Aurora B protein and regulating its stability (Figure 1).

USP48 was previously reported to associate with the COP9 signalosome (CSN) to regulate NF-κB signaling [42]. NF-κB signaling was implicated as a regulator of cell-cycle progression [43,44]; however, we could not find evidence for cell cycle regulatory behavior in USP48. To our knowledge, this is the first report to demonstrate the direct role of USP48 in controlling the cell cycle by regulating Aurora B. Our results demonstrate that the overexpression of USP48 induced the stabilization of both endogenous and exogenous Aurora B proteins, which decreased upon USP48 depletion (Figure 2). We also demonstrated that USP48 and Aurora B proteins co-precipitated with each other. Further, the depletion of USP48 decreased the half-life of Aurora B, while overexpression of USP48 rescued the half-life of Aurora B protein (Figure 3).

Aurora B proteins are regulated by the ubiquitin–proteasome system [27,34,36]. We demonstrated that the overexpression of USP48 counteracted Aurora B ubiquitination and reduced the ubiquitination smear attached to Aurora B protein (Figure 3), consistent with other studies also reporting the active deubiquitination of substrates by USP48 [42,45,46,47]. Additionally, the mRNA expression of *Aurora B* was previously reported to be high in several cancers [13,14,15,16,17,18,19,20,21] which also positively correlated with the expression levels of *USP48* [48,49,50], across a wide panel of cancer cells (Figure 4, Supplementary Figure S1). This positive correlation between USP48 and Aurora B proteins led us to investigate the effect of USP48 depletion on the cell cycle, as Aurora B dysregulation was reported to interfere with the progression of normal mitosis [10,51]. We demonstrated that the depletion of USP48 generated a large number of chromosome segregation errors leading to an increase in the number of multipolar spindles during mitosis (Figure 4). These conditions resulted in the accumulation of multinucleated cells due to USP48 depletion, which is in line with previous reports on Aurora B regulation during cytokinesis [10,36,51,52] (Figure 4 and Figure 5). USP48 depletion also resulted in the downregulation of INCENP, Survivin and CDC25A that are known to be associated with the cell cycle (Figure 5). INCENP and Survivin are an integral part of the CPC complex, which is highly active during mitosis [53], whereas CDC25A proteins control the cell cycle progression through the S phase and entry into mitosis [54]. The potential role of USP48 in regulating individual components of the CPC complex during mitosis needs to be further elucidated.

In conclusion, the DUBKO library kit helped us identify a potential deubiquitinase, USP48, that regulates Aurora B protein levels during the cell cycle. We found that the deubiquitinating activity of USP48 helps to maintain the steady-state levels of Aurora B proteins by regulating their half-life. Depletion of USP48 resulted in mitotic defects leading to cytokinesis failures. Our observations indicate that the interaction between USP48 and Aurora B is critical for faithful mitotic progression by regulating various factors that can influence the cell cycle directly or indirectly and opens up avenues for further investigation.

## 4. Materials and Methods

### 4.1. Plasmids

Full-length human Aurora B was cloned into pcDNA 3.1 6X Myc-vector. HA-tagged ubiquitin (Plasmid #18712), Flag-tagged USP48 (Plasmid #22585), and GFP-H2B (Plasmid #11680) were purchased from Addgene (Watertown, MA, USA). The active cysteine residue at position 98 was replaced with serine, producing USP48C98S (USP48CS) by site-directed mutagenesis and generating the catalytic mutant of USP48. Cas9-2A-mRFP-2A-PAC was purchased from Toolgen (Geumcheon-gu, Seoul, Korea).

### 4.2. Antibodies and Reagents

Mouse monoclonal antibodies against HA (sc-7392, 1:1000), Myc (sc-40,1:1000), β-tubulin (sc-5274, 1:000), USP48 (sc-100635, 1:1000), GAPDH (sc-32233, 1:2000), ubiquitin (sc-8017, 1:1000), and normal mouse IgG (sc-2025, 1:1000) were purchased from Santa Cruz Biotechnology (Dallas, TX, USA). β-Tubulin-Cy3 (C4585, 1:1000) was purchased from Sigma Aldrich (St. Louis, MO, USA). Flag (Anti-DDDDK-tag, M185-3L, 1:1000) was purchased from MBL Life Science (MBL International, Woburn, MA, USA), rabbit polyclonal anti-Cdc25A antibody (55031-1-AP) was purchased from Proteintech (Rosemont, IL, USA), Survivin (Cat. #2803S, Cell Signaling Technology, Danvers, MA, USA) and Aurora B (Cat. # 36-5200, Invitrogen, 1:1000) was purchased from Thermo Fisher Scientific, (Waltham, MA, USA).

In addition, Protein A/G Plus Agarose beads (sc-2003, Santa Cruz Biotechnology, Dallas, TX, USA), protease inhibitor cocktail (Cat. #B14012, Bimake.com, Houston, TX, USA), cycloheximide (CHX; Cat. #C4859, Sigma-Aldrich, St. Louis, MO, USA), RIPA buffer (Cat. #R2002, Bioseong, Gyeonggi-do, South Korea), Protein 5X sample buffer (Cat. #EBA-1052, ELPIS BIOTECH, South Korea), proteasomal inhibitor MG132 (Cat. #S2619, Selleckchem, Houston, TX, USA), Thymidine (Cat. #T9250, Sigma-Aldrich, St. Louis, MO, USA) and Nocodazole (Cat. #M1404, Sigma-Aldrich, St. Louis, MO, USA) were also used.

### 4.3. Cas9 and sgRNA Constructs

For screening DUBs, plasmids encoding single-guide RNAs (sgRNAs) and Cas9-2a-mRFP-2a-PAC (with a puromycin N-acetyl-transferase and puromycin resistance gene) were purchased from Toolgen (Seoul, South Korea). Bioinformatics tools (www.broadinstitute.org, accessed on 3 December 2019) were used to design the sgRNA target sequences, and they were cloned into the vectors as described previously [55]. Briefly, oligonucleotides containing each target sequence were synthesized (Bioneer, Seoul, South Korea), and T4 polynucleotide kinase was used to add terminal phosphates to the annealed oligonucleotides (BioRad, Hercules, CA, USA). Annealed oligonucleotides were ligated into *BsaI*-digested vectors. Oligonucleotide sequences are as follows: sgRNA1 targeting *USP48,* 5′-TCGATGATCCCAACTGTGAG-3′; sgRNA2 targeting *USP48,* 5′-TTTGTGGGCCTGACTAACCT-3′.

### 4.4. Cell Culture and Treatments

Cervical cancer cells (HeLa) and Human embryonic kidney cells (HEK293) (ATCC, Manassas, VA, USA) were cultured in Dulbecco’s Modified Eagle’s medium (DMEM) (GIBCO BRL, Rockville, MD, USA) supplemented with 1% penicillin and streptomycin (GIBCO BRL) and 10% fetal bovine serum (FBS, GIBCO BRL) in a humidified 5% CO_2_ atmosphere at 37 °C. The cells were passaged every 3–4 days using standard cell culture protocols depending on cell confluence. All endogenous and exogenous experiments were performed in HeLa cells and HEK293 cells, respectively. For the cycloheximide (CHX) chase assay, 150 μg/mL CHX (Cat. #C4859, Sigma-Aldrich, St. Louis, MO, USA) was administered, and cells were harvested at the indicated time points (0 h, 0.5 h,1 h, and 2 h).

The plasmids were transfected in HEK293 and HeLa cells using polyethyleneimine (PEI; Polysciences, Warrington, PA, USA) according to the manufacturer’s protocol. The transfected cells were selected after 24 h by incubating with puromycin (1 μg/mL) for 2 days and then passaged until further use. For the endogenous IP and ubiquitination experiments described in Figure 2B,C, respectively, cells were treated with 5 μM MG132 for 8 h prior to harvesting.

### 4.5. Generation of Single-Cell-Derived USP48 Knockout Clones

HeLa cells were co-transfected with plasmids encoding Cas9 and sgRNA1 targeting the *USP48* gene at a 1:2 weight ratio using PEI. HeLa cells were co-transfected with plasmids encoding Cas9 and non-targeted sgRNA (scrambled sgRNA) at a 1:2 weight ratio and further subjected to clonal selection to generate mock control clones. After 24 h post-transfection, successfully transfected cells were selected with puromycin (1 μg/mL) for 48 h. Then, the cells were trypsinized, resuspended in DMEM, and seeded into 96-well plates to establish single-cell-derived clones. Sixteen days after seeding, each well was microscopically evaluated, and single-round colonies were selected. Each selected colony was individually trypsinized and replated into 24-well plates. Seven days after subculture, the single-cell-derived knockout clones were harvested for their genomic DNA and subjected to T7E1 analysis. T7E1-positive clone #10 was expanded and used for further experiments as described. T7E1-negative cells transfected with scrambled sgRNA that showed no disruption of the *USP48* gene were used as controls for all the experiments.

### 4.6. T7 Endonuclease I (T7E1) Assay

The T7E1 assay was performed as previously described [56]. Briefly, genomic DNA isolation was performed using DNeasy Blood and Tissue kits (Qiagen, Hilden, Germany) according to the manufacturer’s instructions. The regions surrounding the nuclease target sites were amplified by PCR using oligonucleotide sequences mentioned in Table 1. PCR-amplified amplicons were denatured by heating to 95 °C and then annealed to form DNA heteroduplexes, which were then treated with 5 units of T7E1 enzyme (New England Biolabs, Ipswich, MA, USA) and incubated for 20 min at 37 °C. DNA fragments were analyzed by 2% agarose gel electrophoresis. The percentage of mutation frequencies was calculated using ImageJ software to quantify band intensities, and the following equation was used: Mutation frequency (%) = 100 × (1 − [1 − fraction cleaved] 1/2), where fraction cleaved is the total relative density of the cleavage bands divided by the sum of the relative density of cleavage and uncut bands.

### 4.7. Immunoprecipitation and Immunoblotting

For exogenous ubiquitination and immunoprecipitation assays, HEK293 cells were harvested and lysed in RIPA buffer (Cat. #RC2002-050-00, Biosesang, Gyeonggi-do, South Korea) containing 50 mM Tris-HCl (pH 7.6), 1% Triton X-100, 150 mM NaCl, 0.1% SDS, 1% sodium deoxycholate, 2 mM EDTA, and 1 mM PMSF after 48–72 h of transfection with their respective constructs. Roughly 2–3 mg of cell lysates were incubated with their respective antibodies at 4 °C overnight followed by immunoprecipitation with 20 μL of protein agarose beads at 4 °C for 2–3 h. The beads were washed with cell lysis buffer containing 150 mM sodium chloride, 1% triton X-100, 1% sodium deoxycholate, 0.1% SDS, 50 mM Tris-HCl (pH 7.6), and 2 mM EDTA followed by elution in 5X denaturing protein sample buffer containing 312.5 mM Tris-HCl (pH 6.8), 50% glycerol, 5% SDS, 5% β-mercaptoethanol, and 0.05% bromophenol blue and denatured by boiling at 95–100 °C for 5 min. These denatured samples were then resolved using 10% SDS-PAGE electrophoresis at 80 V. Resolved bands were transferred to activated PVDF membranes and analyzed by immunoblotting. Images of the immunoblots were captured using the ChemiDoc Imaging System and quantified using the ImageJ software [57].

For endogenous immunoprecipitation assays, HeLa cells were treated with the MG132 proteasomal inhibitor for 8 h prior to harvesting. Roughly 5 mg of lysates were incubated with their respective antibodies and detected using Western blot analysis as described above. Moreover, 3% of the samples were used to identify immunoprecipitation efficiency as input. Mouse IgG (ab-99697, Abcam, Cambridge, MA, USA) and rabbit IgG (CST- 58802S, Cell Signaling Technology, Danvers, MA, USA) light chain-specific secondary antibodies were used to prevent interference from heavy and light immunoglobulin chains in the binding assays.

### 4.8. Time-Lapse Microscopy

USP48KO and mock HeLa cells were transfected with histone H2B-GFP and seeded in a glass-bottom multi-well chamber (four-well chamber, Lab-Tek II chambered cover glass, Thermo Fisher Scientific, Waltham, MA, USA). Time-lapse images were taken at 3 min intervals and maximally projected. Time-lapse live-cell imaging was performed in a CO_2_ chamber at 37 °C (Applied Precision) using a 40X, 1.35NA, 0.10 mm, WD objective lens (Delta Vision Core; GE Healthcare). Data were obtained from three independent experiments.

### 4.9. Immunofluorescence Microscopy

USP48KO and mock HeLa cells were grown on glass coverslips for 48 h and then fixed in 4% paraformaldehyde (Cat. #163-20145, Wako, Richmond, VA, USA) in Phosphate buffered saline (PBS) for 10 min at room temperature. Fixed cells were washed with PBS and permeabilized using 0.1% Triton X-100 (Cat. no. #0694, Amresco, Solon, OH, USA) in PBS for 5 min. Cells were then washed in PBS and blocked for 1 h with 1% BSA (Cat. no. #A9418, Sigma-Aldrich, St. Louis, MO, USA) in PBS. Primary antibodies used for staining were diluted in BSA and incubated overnight at 4 °C. The next day, the cells were incubated with their corresponding conjugated secondary antibodies for 1 h, washed two times in PBS, and mounted onto glass slides. The cells were visualized, and images were captured using a Leica fluorescence microscope (TCS SP5, Leica, Wetzlar, Germany).

### 4.10. Statistical Analysis

For the statistical analyses, GraphPad Prism 9 (GraphPad Software Inc., San Diego, CA, USA) was used, and data are presented as means ± standard deviations considering three independent experiments. Comparison between two groups was carried out using unpaired Student’s *t*-test. Experiments involving three groups or more were analyzed by one-way or two-way analysis of variance (ANOVA) followed by Tukey’s post hoc test. *p*-values less than 0.05 were considered statistically significant.

## Figures and Tables

**Table 1:** Oligonucleotides used to amplify PCR targets for T7E1 assay.

Gene	sgRNA	Step	Direction	Sequence (5′–3′)
*USP48*	sgRNA1	I PCR	FP	CATTTGGGTGGCTTCCAATA
		I PCR	RP	TAAAACAGGCAGCTGCGTAA
		II PCR	FP	TCCACACCCTCACAAACTGA
		II PCR	RP	TAAAACAGGCAGCTGCGTAA
	sgRNA2	I PCR	FP	CGCTTGTTCAAAACCGATCT
		I PCR	RP	TCTGTTTCCCAAGCCAGAGT
		II PCR	FP	TCCTGTGGTCAACCCAAAAT
		II PCR	RP	TCTGTTTCCCAAGCCAGAGT

## Data Availability

All data generated or analyzed during this study are included in this published article and its Supplementary Materials.

## Supplementary Materials

The following are available online at https://www.mdpi.com/article/10.3390/ijms22168508/s1.
