# Supplementary material for: USP48 Governs Cell Cycle Progression by Regulating the Protein Level of Aurora B
Source: Int J Mol Sci. 2021 Aug 7;22(16):8508. doi: 10.3390/ijms22168508 (PMC8395203; doi:10.3390/ijms22168508)
Supplement: Supplementary file 1 [file ijms-22-08508-s001.zip › ijms-1287302 Supplementary Files.pdf]

Supplementary File

USP48 Governs Cell Cycle Progression by Regulating the Protein Level of Aurora B

Ainsley Mike Antao [1,†], Kamini Kaushal [1,†], Soumyadip Das [1], Vijai Singh [2], Bharathi Suresh [1], Kye-Seong Kim [1,3,*] and Suresh Ramakrishna [1,3,*]

[1] Graduate School of Biomedical Science and Engineering, Hanyang University, Seoul 04763, Korea; ainsleyantao@gmail.com (A.M.A.); kaminikaushal10@gmail.com (K.K.); smdpdas6@gmail.com (S.D.); bharathi.suri@gmail.com (B.S.)
[2] Department of Biosciences, School of Science, Indrashil University, Rajpur, Gujarat 382740, India; vijai.singh@indrashiluniversity.edu.in (V.S.)
[3] College of Medicine, Hanyang University, Seoul 04763, Korea
* Correspondence: ks66kim@hanyang.ac.kr (K.-S.K.); suri28@hanyang.ac.kr (S.R.)
† These authors contributed equally.

<u>Table of Contents</u>

Figure S1. Expression box plot showing the differential expression of *Aurora B* and *USP48* in various cancer types

Supplementary methods: Detailed method for transfection and Western blot analysis.

Figure S2. The original uncropped images for all immunoblots represented in the main figures.

Figure S3. The triplicate images for the immunoblots represented as graphs in the main figures.

Supplementary Movie 1. Cytokinesis in mock HeLa cells and USP48 depleted HeLa cells.

Figure S1. Expression box plot showing the differential expression of *Aurora B* and *USP48* in various cancer types

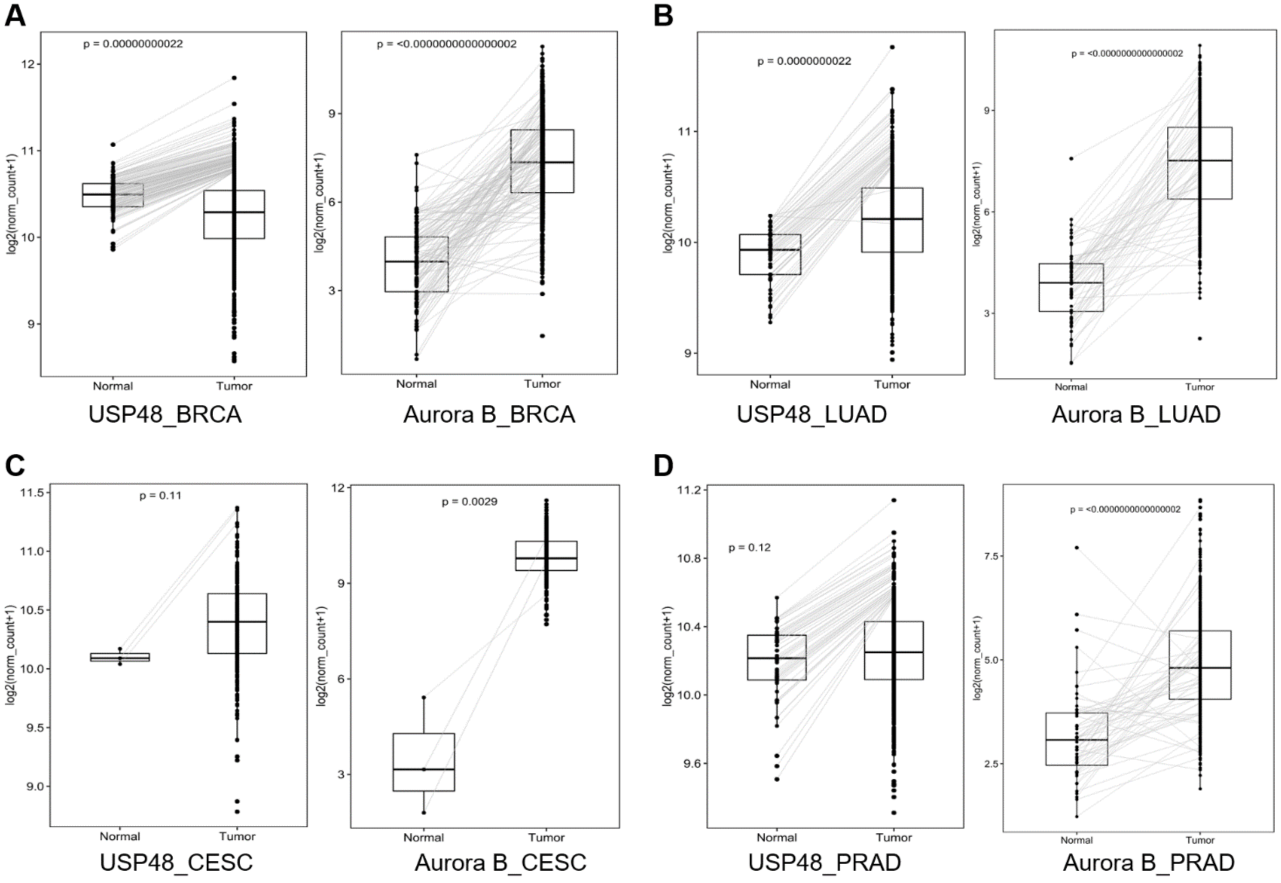

Figure S1: Expression box plot showing the differential expression of *USP48* and *Aurora B* in normal vs. tumor tissues for (**A**) BRCA, (**B**) LUAD, (**C**) CESC, and (**D**) PRAD using RNAseq data from the TCGA database. p value < 0.05 was considered to be statistically significant.

Supplementary methods

Transfection method: HeLa or HEK293 cells were seeded at 2 X 10^5 in 35 mm Cell culture dish or 1.5 X 10^6 in 100 mm Cell culture dish as indicated for each experiment and cultured for about 20 h in a humidified 5% CO_2 atmosphere at 37°C. The indicated concentrations of plasmids and PEI were diluted in Opti-MEM medium at total volume of 200μL for a 35 mm dish or 600 μL for a 100 mm dish. The solution was incubated for 20 minutes at room temperature before adding to each well for transfection. Transfected cells were incubated for 18-20 h prior to media change. The cells were further incubated for 48-72 h for further treatments as indicated or harvested for Western blot analysis.

Immunoblotting method: The harvested cells were lysed in ice-cold RIPA buffer (Cat. #RC2002-050-00, Biosesang, Gyeonggi-do, South Korea) containing 50 mM Tris-HCl (pH 7.6), 1% Triton X-100, 150 mM NaCl, 0.1% SDS, 1% sodium deoxycholate, 2 mM EDTA, and 1 mM PMSF. The lysates were clarified by centrifugation at 15000 rpm for 20 minutes at 4°C followed by protein quantification using a BSA standard by Bradford assay**.** Equal amounts of proteins were aliquoted and diluted using 5X denaturing protein sample buffer containing 312.5 mM Tris-HCl (pH 6.8), 50% glycerol, 5% SDS, 5% β-mercaptoethanol, and 0.05% bromophenol blue and denatured by boiling at 95-100°C for 5 minutes. The denatured samples were then resolved using 10% SDS-PAGE electrophoresis at 80 V and transferred to activated PVDF membranes for 2 h at 0.5 constant amperes. Membranes were blocked for 1 h using 5% skim milk and the respective primary antibodies were diluted in 2.5% skim milk and incubated overnight at 4°C. Immunoblots were washed three times with 1X Tris-buffered saline containing Tween 20 (TBST) and incubated with respective HRP-conjugated secondary antibodies for 1 h at room temperature. Immunoblots were washed three times with TBST and images were captured using the ChemiDoc Imaging System and quantified using the ImageJ software.

Figure S2. The original uncropped images for immunoblots represented in the main figures.

Methodology: Figure 1

Cells seed number: 2 X 10^5 in 35 mm dish for each transfection

Tranfection efficiency: 80-85% transfection

Transfection reagent: 1 μg DNA:2 μg PEI

Figure 1A: Plasmids concentrations: RFP-Cas9 =1 μg; sgRNAs targeting individual USPs/ scrambled sgRNA (mock) =2 μg; Myc-Aurora B=500 ng

Figure 1B: Plasmids concentrations: RFP-Cas9 =1 μg; sgRNAs targeting individual USPs/ scrambled sgRNA (mock) =2 μg

Fig. 1

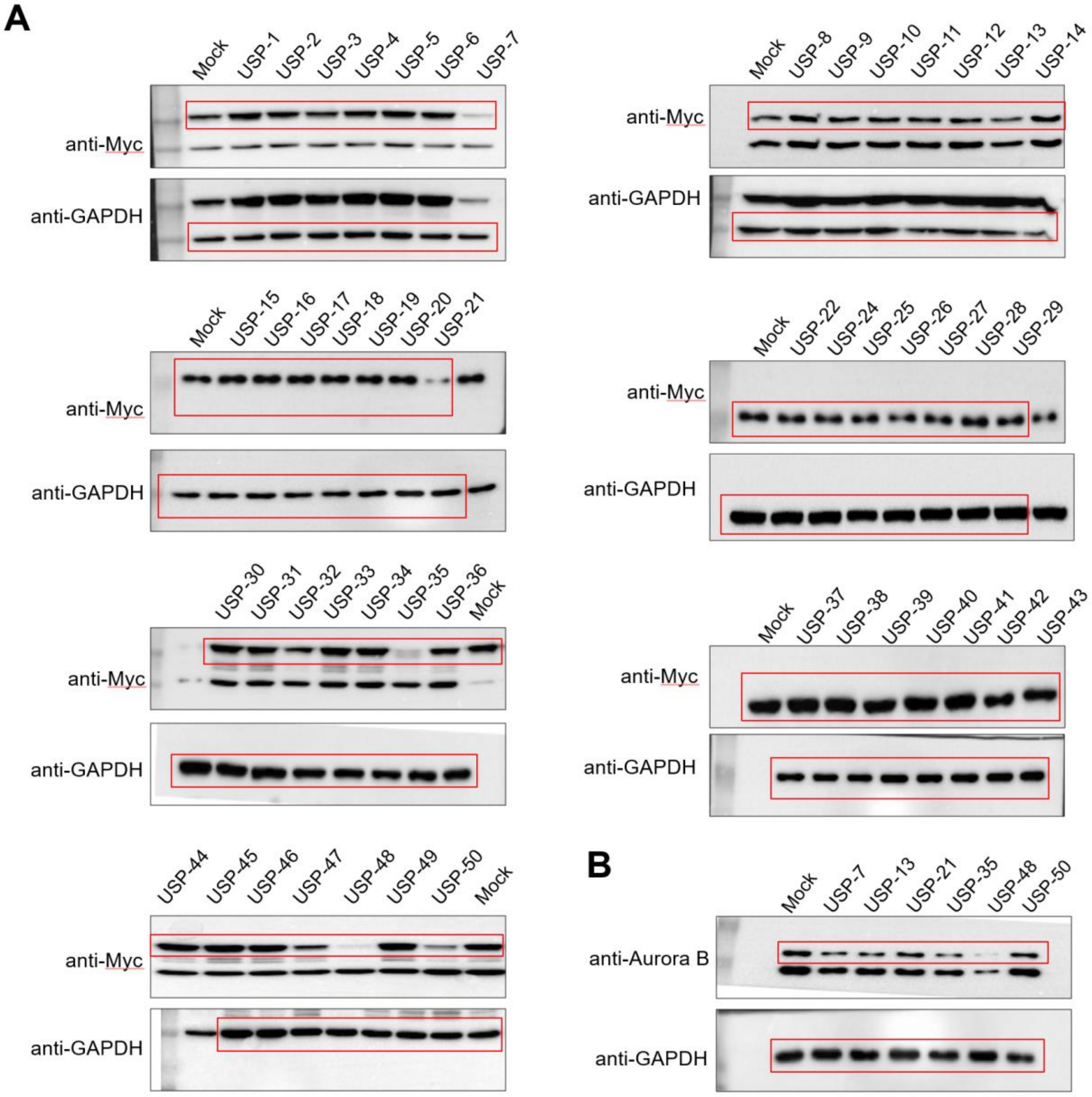

Methodology: Figure 2

Cells seed number: 2 X 10^5 in 35 mm dish for each transfection

Tranfection efficiency: 80-85% transfection

Transfection reagent: 1 µg DNA:2 µg PEI

Figure 2A: Plasmids concentrations: RFP-Cas9 =1 µg; sgRNAs targeting USP48/ scrambled sgRNA (mock) =2 µg

Figure 2B: Plasmids concentrations: RFP-Cas9 =1 µg; sgRNAs targeting individual USPs/ scrambled sgRNA (mock) =2 µg; Myc-Aurora B=500 ng

Figure 2C: Plasmids concentrations: Flag-USP48 = 0, 1, 2, and 3 µg

Figure 2D: Plasmids concentrations: Flag-USP48 = 0, 1, 2, and 3 µg; Myc-Aurora B=500 ng

Fig. 2

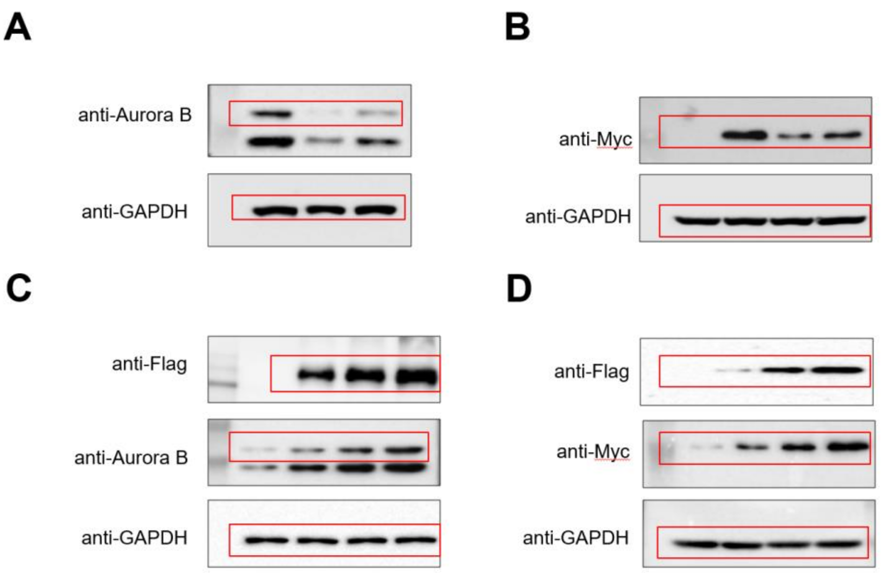

Methodology: Figure 2

Cells seed number: 2 X 10^5 in 35 mm dish for each transfection

Tranfection efficiency: 80-85% transfection

Transfection reagent: 1 µg DNA:2 µg PEI

Figure 2E: Plasmids concentrations: Flag-USP48CS = 0, 1, 2, and 3 µg

Figure 2F: Plasmids concentrations: Flag-USP48CS = 0, 1, 2, and 3 µg; Myc-Aurora B=500 ng

Figure 2G: Plasmids concentrations: RFP-Cas9 =1 µg; sgRNA1 targeting USP48=2 µg; Flag-USP48 = 1 µg

Figure 2H: Plasmids concentrations: RFP-Cas9 =1 µg; sgRNA1 targeting USP48=2 µg; Flag-USP48 = 1 µg; Myc-Aurora B=500 ng

Fig. 2

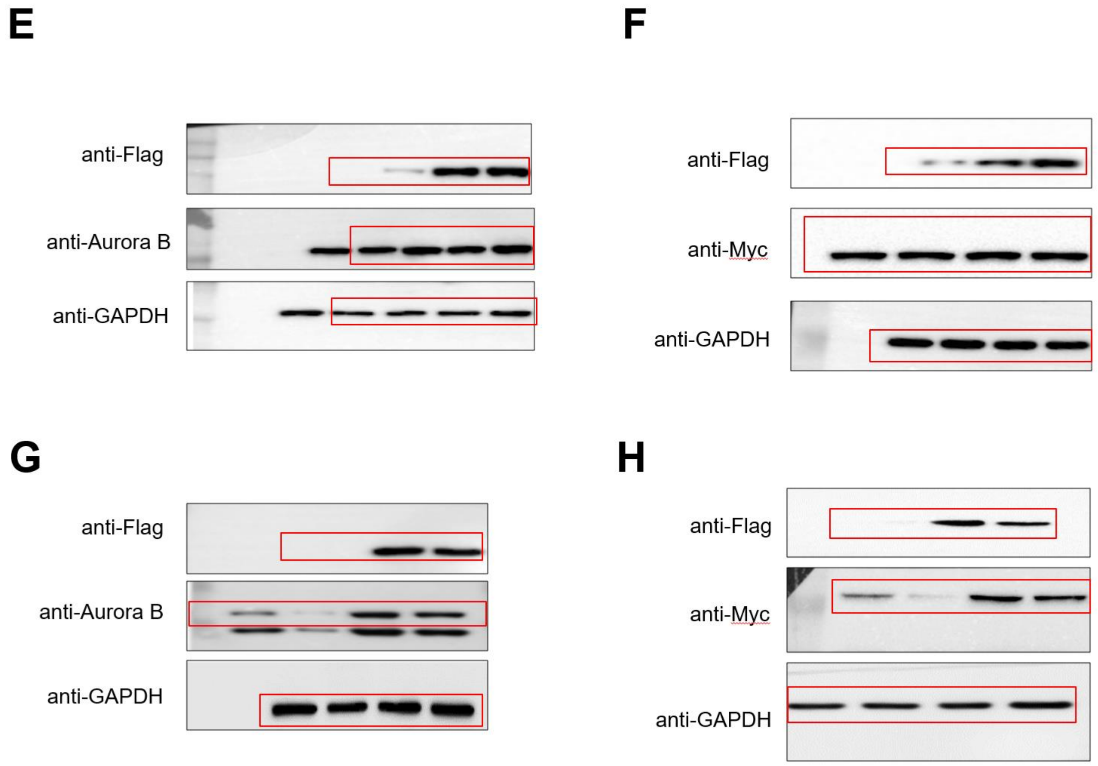

Methodology: Figure 3

Cells seed number: 1.5 X 10^6 in 100 mm dish for each transfection

Tranfection efficiency: 80-85% transfection

Transfection reagent: 1 µg DNA:2 µg PEI

Figure 3A: Reagent for cell treatment: MG132 (5 µg) for 8hr

Figure 3B: Plasmids concentrations: Flag-USP48 = 2 µg; Myc-Aurora B = 2 µg

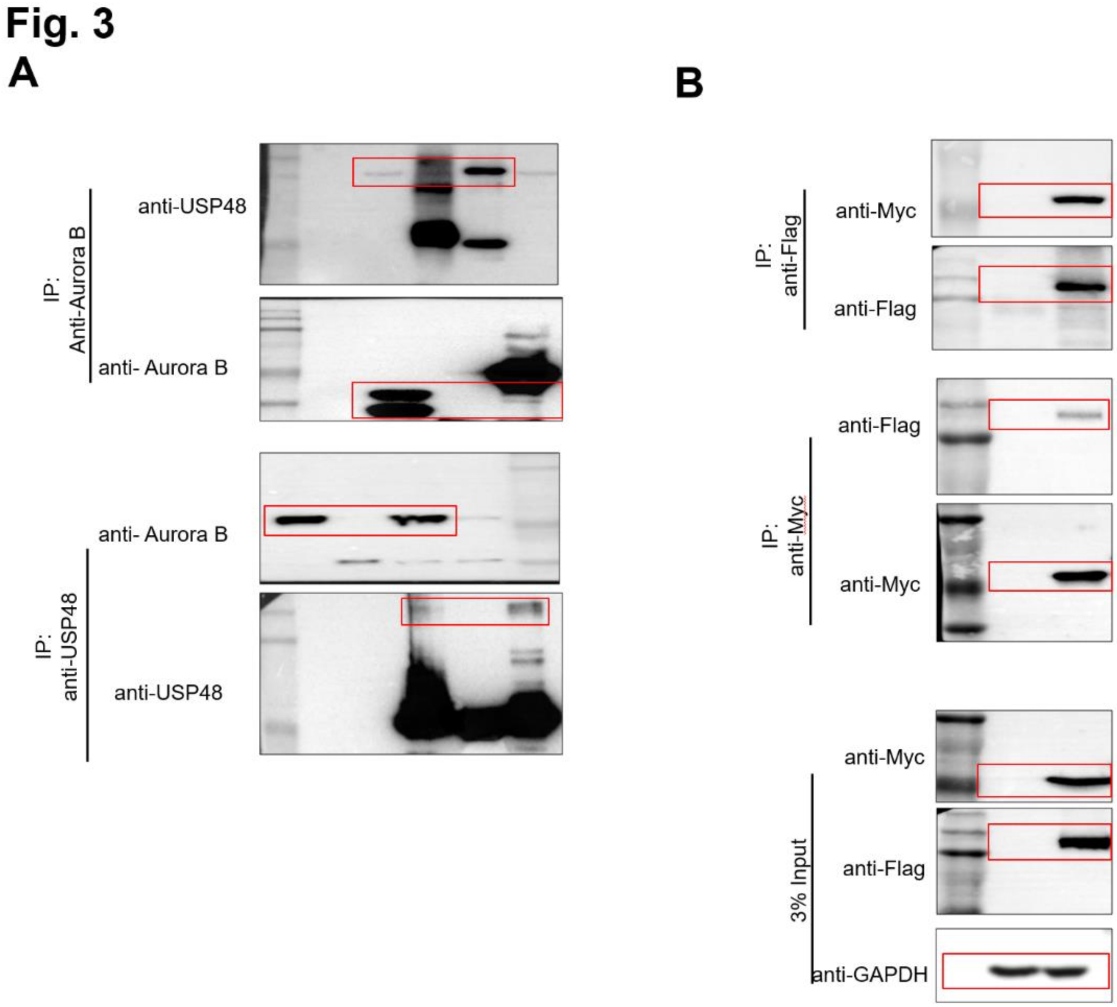

Methodology: Figure 3

Cells seed number: 1.5 X 10^6 in 100 mm dish for each transfection

Tranfection efficiency: 80-85% transfection

Transfection reagent: 1 μg DNA:2 μg PEI

Figure 3C: Plasmids concentrations: Flag-USP48 = 2 μg; Flag-USP48CS = 2 μg; HA-Ubiquitin = 2 μg; Myc-Aurora B = 2 μg. Reagent for cell treatment: MG132 (5 μg) for 8hr

Figure 3D: Cells seed number: 2 X 10^5 in 35 mm dish for each transfection. Plasmids concentrations: RFP-Cas9 =1 μg; sgRNA1 targeting USP48 = 2 μg; Flag-USP48 = 1 μg

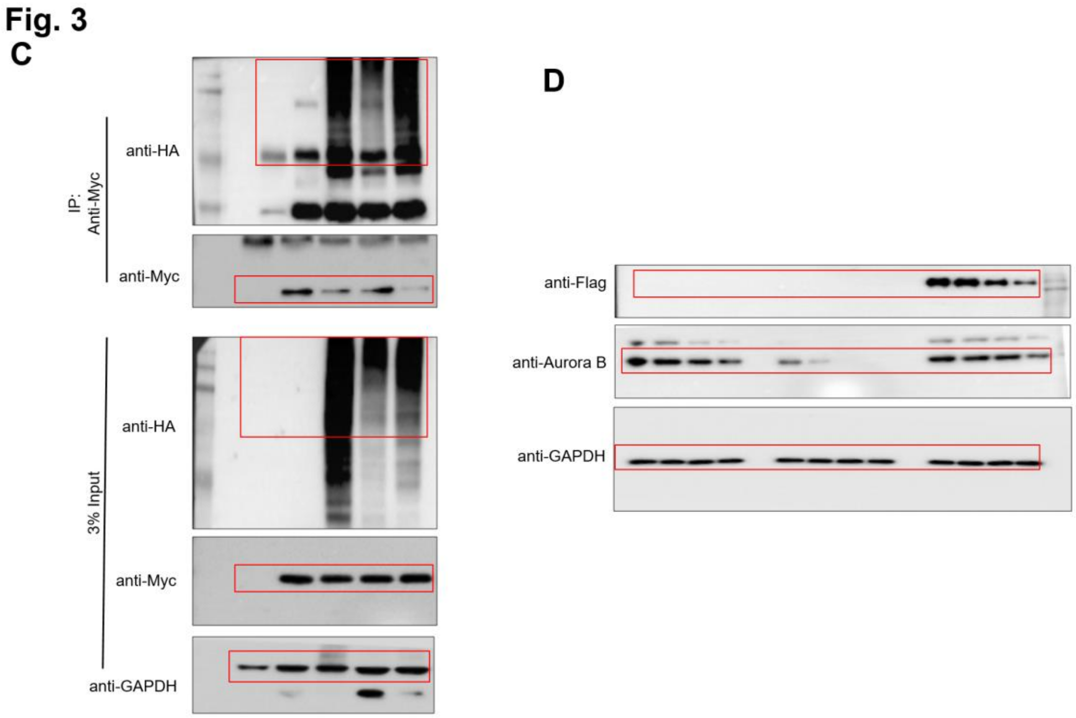

Methodology: Figure 4B

Cells seed number: 2 X 10^5 in 35 mm dish

Reagent for cell treatment: 100 ng/mL Nocodazole for 18 h

Fig. 4

B

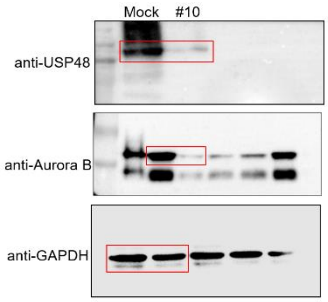

Methodology: Figure 5D

Cells seed number: 2 X 10^5 in 35 mm dish for each transfection

Tranfection efficiency: 80-85% transfection

Transfection reagent: 1 μg DNA:2 μg PEI

Plasmids concentrations: Flag-USP48 = 1 μg; Flag-USP48CS = 1 μg

Fig. 5

D

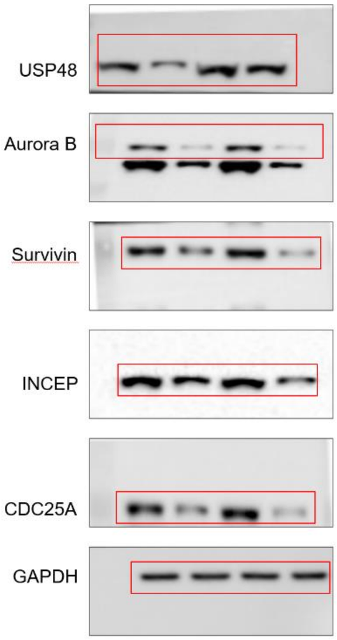

Figure S3. The triplicate images for the immunoblots represented as graphs in the main figures.

Figure 2:

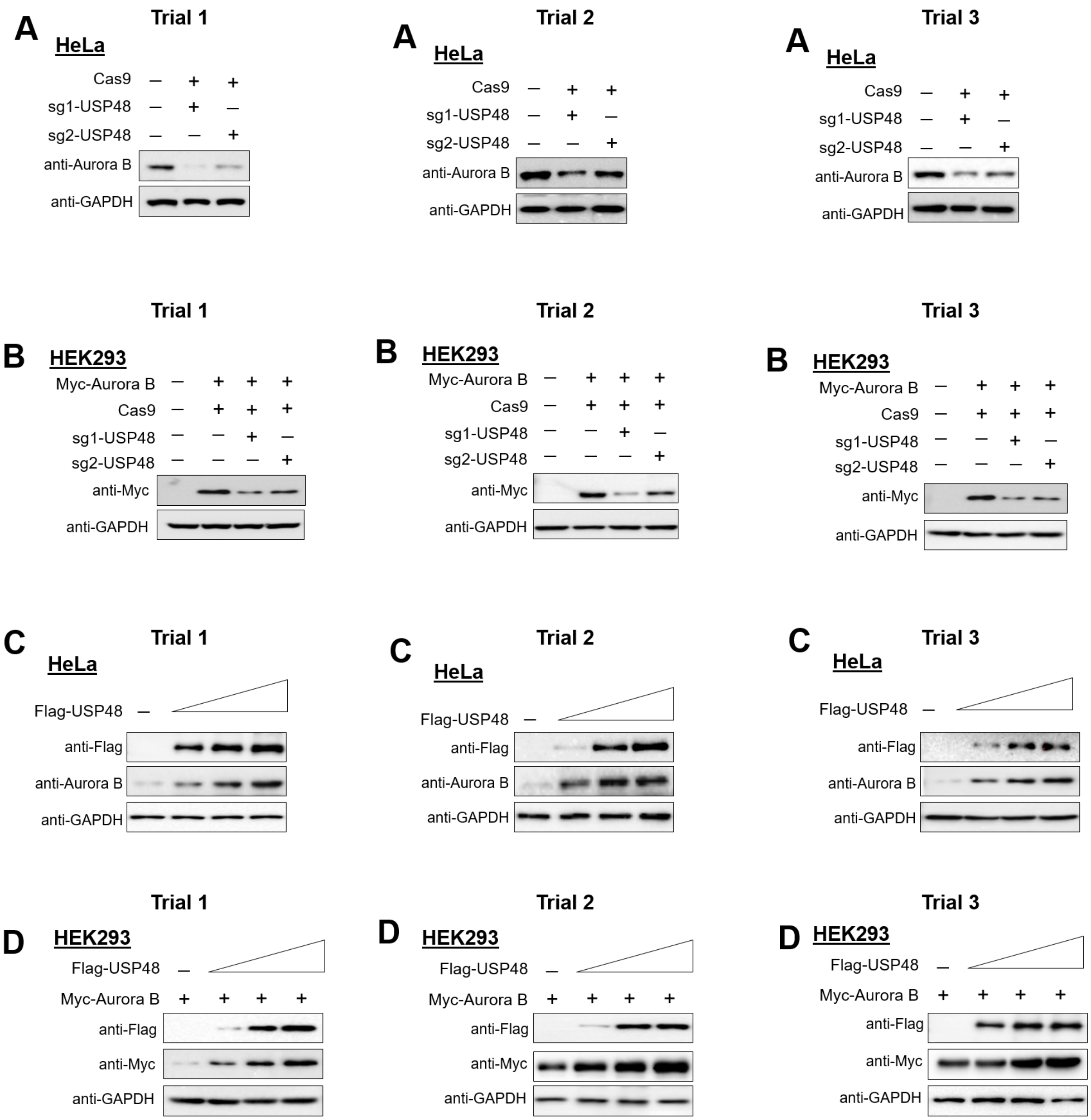

Figure 2:

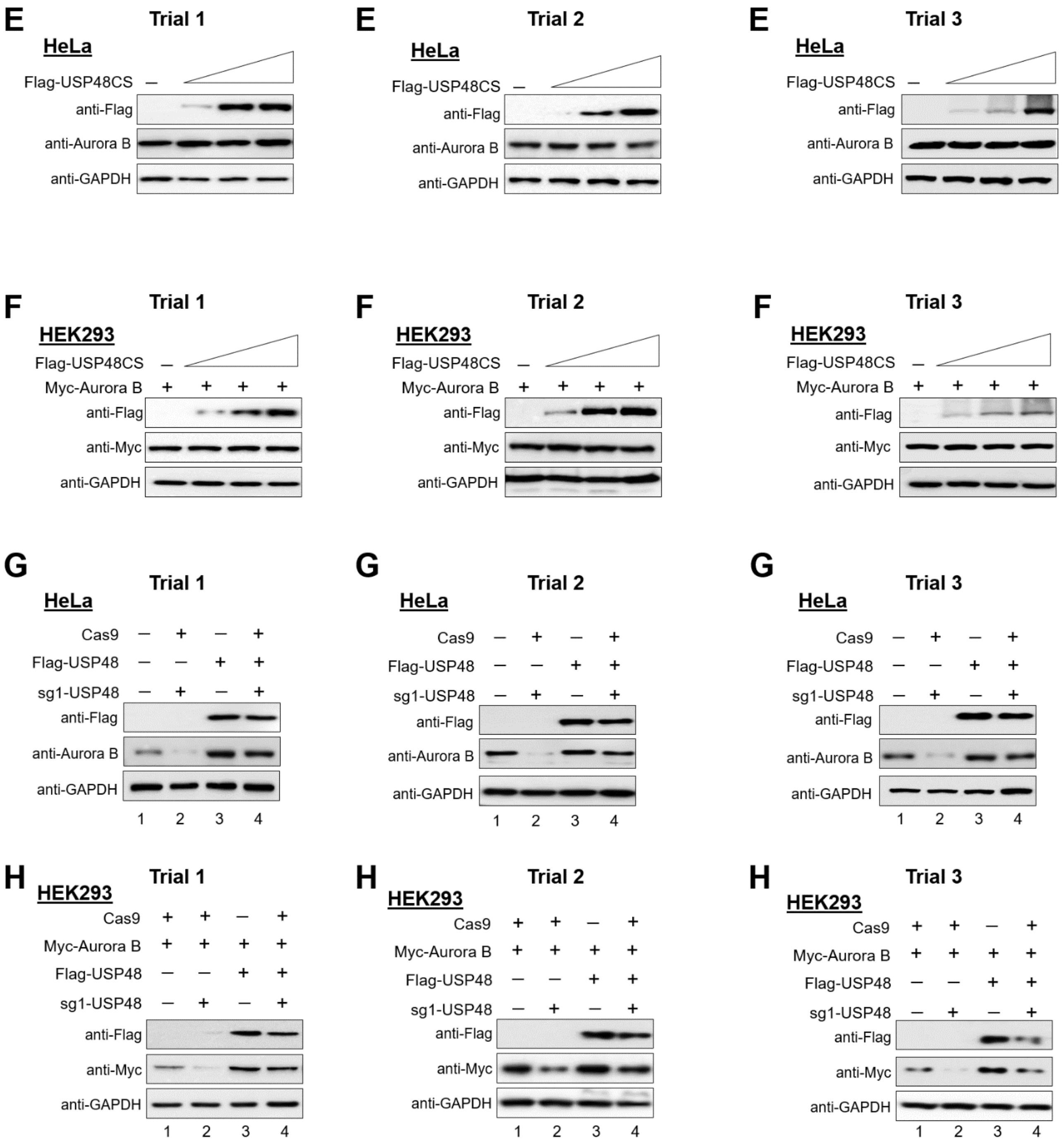
E
Trial 1
HeLa
Flag-USP48CS
anti-Flag
anti-Aurora B
anti-GAPDH
E
Trial 2
HeLa
Flag-USP48CS
anti-Flag
anti-Aurora B
anti-GAPDH
E
Trial 3
HeLa
Flag-USP48CS
anti-Flag
anti-Aurora B
anti-GAPDH
F
Trial 1
HEK293
Flag-USP48CS
Myc-Aurora B
anti-Flag
anti-Myc
anti-GAPDH
F
Trial 2
HEK293
Flag-USP48CS
Myc-Aurora B
anti-Flag
anti-Myc
anti-GAPDH
F
Trial 3
HEK293
Flag-USP48CS
Myc-Aurora B
anti-Flag
anti-Myc
anti-GAPDH
G
Trial 1
HeLa
Cas9
Flag-USP48
sg1-USP48
anti-Flag
anti-Aurora B
anti-GAPDH
1 2 3 4
G
Trial 2
HeLa
Cas9
Flag-USP48
sg1-USP48
anti-Flag
anti-Aurora B
anti-GAPDH
1 2 3 4
G
Trial 3
HeLa
Cas9
Flag-USP48
sg1-USP48
anti-Flag
anti-Aurora B
anti-GAPDH
1 2 3 4
H
Trial 1
HEK293
Cas9
Myc-Aurora B
Flag-USP48
sg1-USP48
anti-Flag
anti-Myc
anti-GAPDH
1 2 3 4
H
Trial 2
HEK293
Cas9
Myc-Aurora B
Flag-USP48
sg1-USP48
anti-Flag
anti-Myc
anti-GAPDH
1 2 3 4
H
Trial 3
HEK293
Cas9
Myc-Aurora B
Flag-USP48
sg1-USP48
anti-Flag
anti-Myc
anti-GAPDH
1 2 3 4

Figure 3:

D

Trial 1

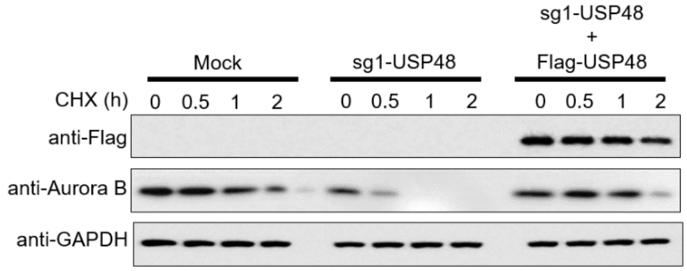

D

Trial 2

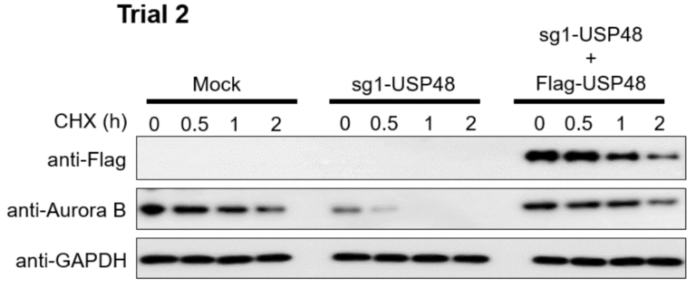

D

Trial 3

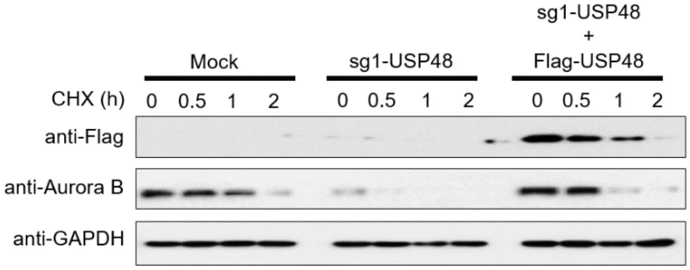

Supplementary Movie 1. Cytokinesis in mock HeLa cells and USP48 depleted HeLa cells. The video clips are attached separately.
